# A large-scale plasma proteome Mendelian randomization study identifies novel causal plasma proteins related to primary biliary cholangitis

**DOI:** 10.3389/fimmu.2023.1052616

**Published:** 2023-02-07

**Authors:** Hongqun Yang, Lanlan Chen, Yahui Liu

**Affiliations:** Hepatobiliary and Pancreatic Surgery Department, General Surgery Center, First Hospital of Jilin University, Changchun, Jilin, China

**Keywords:** primary biliary cholangitis, plasma protein, ficolin-1 (FCN1), CD40, FAM177A1, biomarker

## Abstract

**Background and aims:**

Primary biliary cholangitis (PBC) is a progressive chronic autoimmune cholestatic liver disease characterized by the destruction of small intrahepatic bile ducts leading to biliary cirrhosis. Liver biopsy is required in the diagnosis of Antimitochondrial antibody-negative patients. Therefore, novel biomarkers are needed for the non-invasive diagnosis of PBC. To identify novel biomarkers for PBC, we conducted large-scale plasma proteome Mendelian randomization (MR).

**Methods:**

A total of 21,593 protein quantitative trait loci (pQTLs) for 2297 circulating proteins were used and classified into four different groups. MR analyses were conducted in the four groups separately. Furthermore, the results were discovered and replicated in two different cohorts of PBC. Colocalization analysis and enrichment analysis were also conducted.

**Results:**

Three plasma proteins (ficolin-1, CD40 and protein FAM177A1) were identified and replicated as being associated with PBC. All of them showed significant protective effects against PBC. An increase in ficolin-1 (OR=0.890 [0.843-0.941], p=3.50×10^-5^), CD40 (OR=0.814 [0.741-0.895], p=1.96×10^-5^) and protein FAM177A1 (OR=0.822 [0.754-0.897], p=9.75×10^-6^) reduced the incidence of PBC. Ficolin-1 (PP4 = 0.994) and protein FAM177A1 (PP4 = 0.995) colocalized with the expression of the genes FCN1 and FAM177A1 in whole blood, respectively. Furthermore, CD40 (PP4 = 0.977) and protein FAM177A1 (PP4 = 0.897) strongly colocalized with PBC.

**Conclusions:**

We expand the current biomarkers for PBC. In total, three (ficolin-1, CD40, and protein FAM177A1) plasma proteins were identified and replicated as being associated with PBC in MR analysis. All of them showed significant protective effects against PBC. These proteins can be potential biomarkers or drug targets for PBC.

## Introduction

Primary biliary cholangitis (PBC) is a progressive chronic autoimmune cholestatic liver disease characterized by the destruction of small intrahepatic bile ducts leading to biliary cirrhosis (1). A systematic review of epidemiological studies suggested that the PBC incidence ranges from 0.3 to 5.8 per 1000 people and that prevalence rates are increasing over time (2). Although the specific etiology of PBC remains uncertain, several triggers have been identified by previous studies. The immunogenetic risk and epigenetic regulation of the epithelium and bile acid play important roles in the etiology of PBC (3). Immune-mediated biliary injury and the consequences of chronic cholestasis are the major pathogenic features of PBC. The antimitochondrial antibody (AMA) and alkaline phosphatase (ALP) are the main serological biomarkers for the diagnosis of PBC. Although AMA is crucial for the diagnosis of PBC, approximately 3%-5% of patients are AMA negative (4). Therefore, novel biomarkers are required to diagnose AMA-negative PBC and serve as auxiliary biomarkers for AMA-positive PBC. Recently published studies have identified several novel biomarkers for PBC. Bombaci et al. tested 1658 human plasma proteins and found that SPATA31A3 and GARP showed high reactivity in PBC sera (4). A multivariate analysis showed that an elevated level of immunoglobulin M contributes to the diagnosis for patients with seropositive AMA but normal ALP (5). Two retrospective studies conducted by Hayashi suggested that high serum levels of cytokeratin-16 fragment M30, growth arrest-specific gene 6 protein and Axl were associated with the cirrhosis condition of PBC patients (6, 7). Anti-Sp100 and anti-gp210 were identified to be related to PBC (1). Although several serum biomarkers were found, more molecules have not been tested.

Plasma proteins play key roles in a series of biological processes, including signaling, transportation, and inflammation (8). Plasma proteins can originate from any organ, cell, or even from the mother through the placenta (9). Therefore, they could serve as an important source of biomarkers (8). Recently, several genome-wide association studies (GWASs) of plasma proteins have identified protein quantitative trait loci (pQTLs) for thousands of plasma proteins (10–18). A pQTL is an association of protein levels at a genetic locus and is represented by the strongest associating single-nucleotide polymorphism (SNP) (8). Plasma pQTLs represent the circulating levels of plasma proteins. They provide an opportunity for us to test the causal effects of plasma proteins on PBC. To evaluate the causal effects of plasma proteins on PBC and to determine potential biomarkers (risk and protective proteins against PBC), we carried out a large-scale plasma proteome Mendelian randomization (MR) using plasma pQTLs as instrumental variables.

MR is a powerful method to detect the causal effect of exposure (plasma proteins) on the outcome (PBC) using genetic variants extracted from GWAS summary statistics as instrumental variables. Two-sample MR can calculate the causal effect of exposure on outcome using genetic variants that are only associated with exposure and affect outcome through exposure only. Compared to conventional randomized controlled trials, MR is more appropriate to detect a long-term causal effect of risk/protective factors on the outcome due to the random assortment and lifelong effect of genetic variants. Compared to observational studies, MR could avoid environmental confounders and reverse causality because genetic variants used in MR cannot be easily modified by the environment (19). Furthermore, the high efficiency and low cost make MR more suitable for large-scale screening for causal relationships. As a result, we conducted two-sample MR analyses for the plasma proteome using pQTLs extracted from nine different GWASs (10–18). In this study, to eliminate bias, MR analyses were conducted using different types of pQTLs. Furthermore, to reduce chance findings, all proteins were discovered and validated in two different cohorts of PBC. Only proteins identified and validated in two different cohorts were included in our results. Although MR is a powerful tool for detecting causal effects, the results can be confounded by linkage disequilibrium (LD). When exposure and outcome were affected by two different genetic variants that are in LD with each other, we obtained false positive results. Therefore, to eliminate potential LDs, colocalization analyses between proteins and PBC were conducted. Colocalization can determine whether two traits share causal variants in a single region. If the colocalization results suggest strong evidence that exposure and outcome have distinct causal variants in a single region, the MR result is invalid and is removed from the results. Furthermore, to test the source of plasma protein, colocalization analyses between expression quantitative trait loci (eQTLs) and pQTLs were conducted. The proteins identified by MR and colocalized with PBC are more likely to be drug targets (20). Finally, pathway enrichment analysis was conducted to determine the pathways involved in the pathogenesis of PBC. The enriched pathways imply the molecular basis of the causal effects of the plasma proteins on PBC.

This analysis aims to evaluate the causal effect of plasma proteins on PBC and to identify potential biomarkers for PBC.

## Method

**Figure 1:**
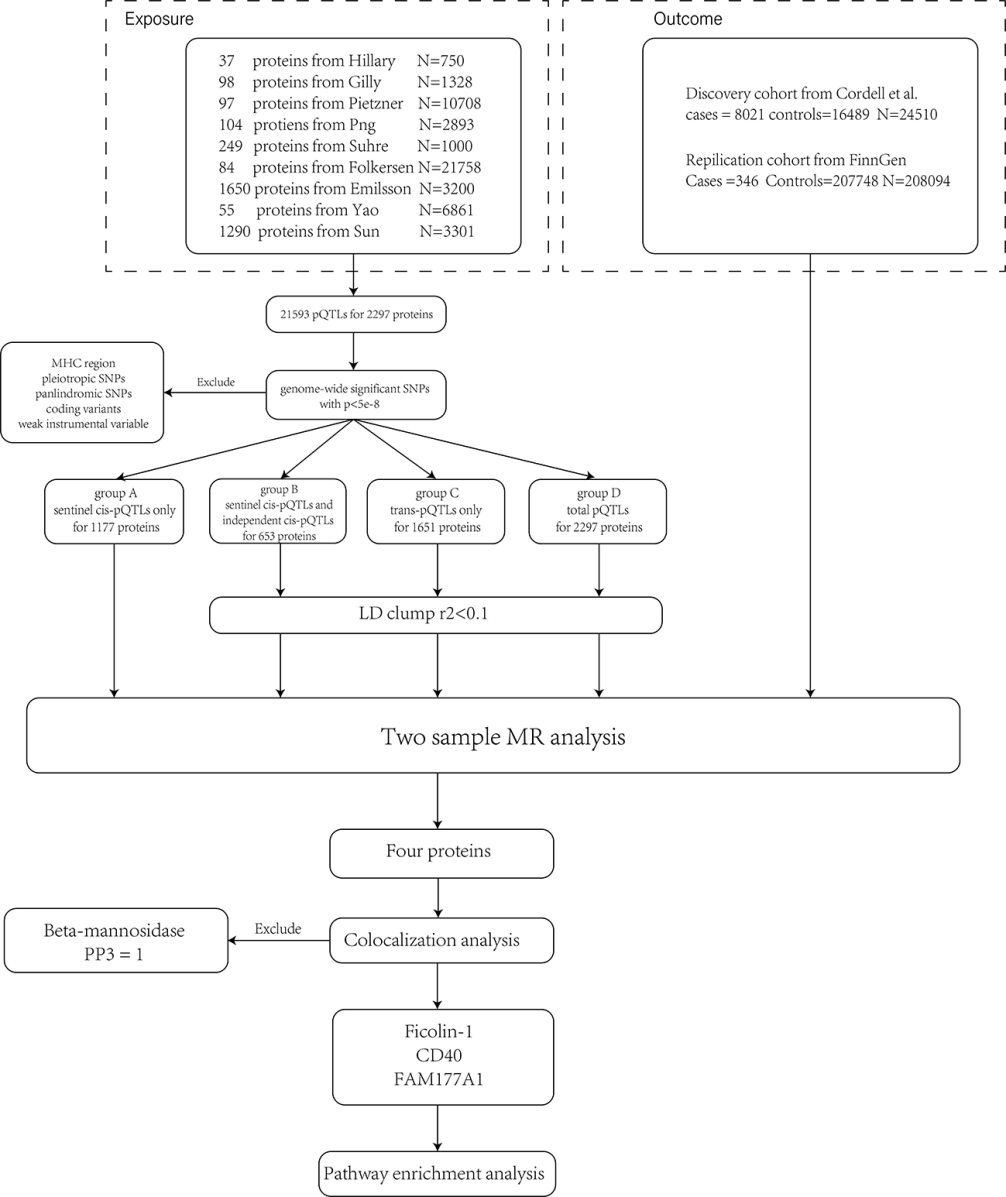
The flow chart shows the analysis process. First, we pooled pQTLs from nine different studies together and removed SNPs violating the MR assumptions. Second, we divided pQTLs into four groups and conducted MR analyses separately. Since sentinel cis-pQTL is the most significant SNP in a region, group A was not LD clumped. Four proteins were identified and validated in the two cohorts. The colocalization analysis suggested that beta-mannosidase and PBC have different causal signals in a single region (PP3 = 1). Therefore, it violated the assumptions of MR and was excluded from the result. Enrichment analysis was conducted on the other three proteins.

As described in the previous section, a large-scale plasma proteome MR analysis was carried out. The process is shown in **Figure 1**.

### Data source

We extracted summary statistics of pQTLs for plasma proteins from nine different proteomic GWASs and pooled them together using METAL. (10–18, 21) In total, 51,799 individuals were included in our analysis. All of the participating individuals are of European ancestry. There is no overlap among the nine GWASs. Details of these studies are provided in ST1.

The summary statistics of PBC were extracted from two different cohorts: a discovery cohort and a replication cohort. Only the proteins that were significant in the discovery cohort and replicated in the replication cohort were considered to be associated with PBC. The discovery summary statistics were obtained from the research of Cordell et al. (22) A total of 8021 European ancestry cases and 16,489 European ancestry controls participated in this GWAS. We extracted summary statistics from the FinnGen cohort (https://r6.finngen.fi/pheno/CHIRBIL_PRIM ) as replication. This included 346 cases and 207,748 controls of European ancestry. Although the participants of the FinnGen cohort were from nine different cohorts (https://finngen.gitbook.io/documentation/methods/cohort-description ), the proportion of cases is relatively small. Since only the participants of the FinnGen cohort did not overlap with the discovery cohort, the FinnGen cohort was the only choice for replication.

All of the GWAS summary statistics adopted in this study are publicly available and freely downloadable. Ethics approval was obtained by the original analysis.

### Instrumental variable selection

pQTLs chosen for the MR analysis must meet the three assumptions of the IV (1): the IV is associated with the risk factor (2); the IV is not associated with confounders; and (3) the IV influences outcome only through the risk factor (23).

To ensure assumption one, only the genome-wide significant (p<5×10^-8^) pQTLs were selected as IVs. Moreover, IVs with an F statistic of less than 10 were regarded as weak IVs and were excluded from this study. As described in assumptions two and three, pQTLs from the MHC region (chr6:27477797-34448354 hg19), palindromic SNPs, and pleiotropic SNPs associated with more than 5 proteins were excluded from this study. To further avoid pleiotropy, MR-PRESSO tests were conducted to identify and remove SNPs with pleiotropy (24). Since the coding variants may affect the assessment of proteins, we removed them from this study (8). pQTLs from Hillary, Suhre, and Sun did not provide a predicted consequence. Therefore, we looked up their consequences using Variant Effect Predictor (25).

To further avoid bias, we divided the pQTLs into four groups: sentinel cis-pQTLs only, sentinel cis-pQTLs combined with independent cis-pQTLs, trans-pQTLs only, and total pQTLs. Then, we named them groups A, B, C, and D, respectively. The pQTL with the lowest p value in a region was selected as the sentinel pQTL. Independent pQTLs were identified by conditional analysis using COJO (26). Due to the lack of pleiotropy and the direct relationship with exposure, the result of sentinel cis-pQTLs only (group A) was preferred in our analysis, and group B was the second choice. Although trans-acting pQTLs (group C) may be pleiotropic, we removed pleiotropic pQTLs. The results of group C could provide a way of understanding the potential etiology of PBC. The results of all pQTLs (group D) could reflect the total causal effect of exposures on outcome. Except for sentinel cis-pQTLs, the other three groups were LD clumped (r2<0.1) because the sentinel cis-pQTL is the most significant SNP in a region.

### Mendelian randomization

MR analyses were carried out in each group. The Wald ratio was adopted in single IV MR. The result of the inverse-variance weighted regression model (IVW) was adopted as the main result. If heterogeneity was detected, the multiplicative effects of IVW were chosen for the result; otherwise, fixed effects of IVW were preferred. In addition, Egger’s regression and the weighted median were also conducted as references. To test the right causal direction, MR Steiger was carried out. For the sake of the robustness of the result, the leave-one-out sensitivity test and heterogeneity test were also performed. We calculated the MR statistical power using mRnd (https://cnsgenomics.shinyapps.io/mRnd/ ) (27). The false discovery rate (FDR) was adopted to adjust the multiple testing. Proteins with FDR <0.05 indicated a causal effect on PBC. Only the proteins that were significant in the discovery cohort and replicated in the replication cohort were considered to be associated with PBC.

### Colocalization analysis

Colocalization analysis was carried out to test whether two traits shared causal variants in a single region of the genome. It was assumed that there was only one causal variant in the region per trait. Furthermore, samples should be from the same ethnic group (28). Based on the single causal variant assumption, we can classify situations into four hypotheses: H0, no causal variants for either trait; H1, a causal variant for trait 1; H2, a causal variant for trait 2; H3, two different causal variants for trait 1 and trait 2; and H4, a shared causal variant between two traits. First, the approximate Bayes factors were calculated using effect estimates and standard errors of each SNP. Then, the log Bayes factors of each hypothesis were calculated. Finally, the posterior probability (PP) for each hypothesis was calculated using Bayes factors and prior probabilities. The hypothesis with PP>80% is likely to be true. In this study, two-trait and multitrait colocalization analyses were conducted.

First, colocalization analyses were conducted between eQTLs and pQTLs within the region of a single gene. The eQTLs were obtained from the gtex portal (https://www.gtexportal.org/home/ ). On account of those genes of eQTLs encoding plasma proteins, we extracted the eQTLs of whole blood. The pQTL is the sentinel pQTL in a single region. By doing so, we were able to determine whether gene expression and protein expression are relevant in whole blood. If eQTLs colocalized with pQTLs, the protein could be generated from whole blood. Then, to eliminate the potential LD and replicate the MR results, colocalization analyses between sentinel cis-pQTLs and PBC were carried out. Because of the larger number of cases, summary statistics of the discovery cohort were adopted in a two-trait colocalization analysis. If there is strong evidence that a protein and PBC have distinct causal variants in a gene region (PP of H3 ≈1), it can be inferred that the positive finding of MR is confounded by LD and will be excluded from this study (29). If a shared variant is found between a protein and PBC, the causal effect of the protein on PBC is reinforced. SNPs within 2 Mb of the sentinel cis-pQTL were analyzed. Proteins identified using MR and colocalized with PBC are likely to be drug targets (20).

### Enrichment analysis

To determine the pathways overrepresented in proteins, we performed enrichment analyses using Reactome (30, 31). All significant proteins were included in this analysis. Based on the effect direction, the proteins were divided into the following two groups: OR>1 and OR<1. These groups underwent enrichment analysis separately. By doing this, we could identify pathways that have more target proteins. The results were corrected for a FDR.

All analyses and data visualization were performed using METAL and R version 4.1.2. The R packages ‘TwoSampleMR’, ‘MRPRESSO’, ‘coloc’, ‘moloc’, ‘ggplot2’, and ‘locuscomparer’ were employed in this study.

## Results

Generally, ten different plasma proteins showed causal effects on PBC in the discovery cohort, and four (ficolin-1, beta-mannosidase, CD40, and protein FAM177A1) of them were replicated in the replication cohort. Among the four proteins, colocalization analyses suggested that beta-mannosidase and PBC have distinct causal variants in a single region. As mentioned previously, this condition violates the MR assumptions, and beta-mannosidase was excluded from our result. The other three proteins showed protective effects on PBC. Ficolin-1 and protein FAM177A1 colocalized with eQTLs from whole blood. Furthermore, CD40 and protein FAM177A1 strongly colocalized with PBC.

As shown in **Figure 1**, we pooled 21,593 pQTLs for 2297 proteins in this study. The full list of proteins is provided in ST2-5. As mentioned previously, we divided pQTLs into four groups. Due to the potential pleiotropy of trans-pQTLs, the results of cis-pQTLs, especially sentinel cis-pQTLs, were preferred in this study. If proteins were identified in multiple groups, the result of group A is shown in the main text. The full list of results is provided in ST10. Finally, after the strict scrutinization for IVs, 460, 454, 541, and 874 proteins were included in groups A, B, C, and D, respectively. Detailed information on plasma proteins and corresponding SNPs are listed in ST2-5. Using the above proteins, MR analyses were conducted on the discovery cohort and replication cohort simultaneously.

### MR results

The full results of the discovery cohort are provided in ST6-9. A total of 10 different circulating plasma proteins showed causal effects on PBC in the discovery result. The details are listed in ST10. The following four proteins also showed a significant causal effect in the replication results: ficolin-1, beta-mannosidase, CD40, and protein FAM177A1. Except for beta-mannosidase, which violates the MR assumptions (detected by colocalization analysis and discussed in the next section) and was excluded from the results, three causal proteins were found in this study. As shown in **Table 1**, all of them showed protective effects against PBC. Ficolin-1 was identified in group A, while CD40 showed a significant causal effect in both groups A and B. Protein FAM177A1 was identified in groups B and D. None of the four proteins were identified in group C (trans-pQTL only), suggesting that there was little pleiotropy in our analyses. Circulating CD40 (OR=0.814 [0.741-0.895], p=1.96×10^-5^) showed a significant protective effect on PBC in the two groups of cis-pQTLs. The incidence of PBC was reduced per 1-SD increase in ficolin-1 (OR=0.890 [0.843-0.941], p=3.50×10^-5^). Both the IVW result (p=3.50×10^-5^) and the weighted median result (p=3.21×10^-4^) were significant, indicating the robustness of our result. A 1-SD increase in the protein FAM177A1 (OR=0.822 [0.754-0.897], p=9.75×10^-6^) reduced the incidence of PBC.

**Table 1:** MR results for significant proteins in the discovery cohort that were replicated in the replication cohort.

protein	UniprotID	method	pval	FDR	OR	lower	upper	r2	power	group
ficolin-1	O00602	Inverse variance weighted (fixed effects)	3.50E-05	3.24E-03	0.890	0.843	0.941	0.278	0.99	group A
CD40	P25942	Wald ratio	1.96E-05	2.27E-03	0.814	0.741	0.895	0.096	0.99	group A and group B
protein FAM177A1	Q8N128	Wald ratio	9.75E-06	1.67E-03	0.822	0.754	0.897	0.111	1	group B and D

Method: the method adopted for the protein. OR is the odds ratio. P value is the p value of the odds ratio. FDR is the p value adjusted using the false discovery rate for multiple tests. The 95% LCI is the lower limit of the 95% confidence interval for the OR. The 95% UCI is the upper limit of the 95% confidence interval for the OR. R2 is the variance explained by genetics. Power is the statistical power calculated using mRnd. Group is the IV group. If a protein showed a significant effect in multiple groups, the result of group A is presented in the table.

### Colocalization analysis

**Figure 2:**
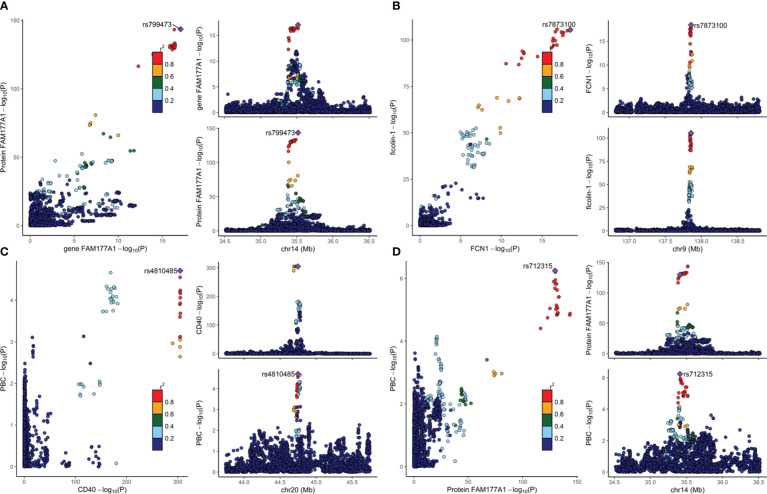
The colocalization results are visualized in **Figure 2**. The lead SNP is indicated by the purple diamond. Strong evidence of colocalization was shown. The plots on the right show the causal variant of two traits from the same locus. The -log P values of two traits from a single locus are plotted on the left. **(A)** Colocalization analysis of eQTLs for gene FAM177A1 and pQTLs for protein FAM177A1. **(B)** Colocalization analysis of eQTLs for gene FCN1 and pQTLs for ficolin-1. **(C)** Colocalization analysis for pQTLs of CD40 and PBC. **(D)** Colocalization analysis of pQTLs for protein FAM177A1 and PBC.

As discussed previously, colocalization analyses between eQTLs from whole blood and pQTLs were performed. We mapped the UniProt ID to the ensemble ID (ST11). As shown in **Figures 2A, B**, the expression of the proteins FAM177A1 (PP for H4 (PP4) =0.995) and ficolin-1 (PP4 = 0.994) colocalized strongly with the expression of the genes FAM177A1 and FCN1 (encoding ficolin-1) in whole blood, respectively. The causal variant can regulate both gene and protein expression. It can be inferred that the proteins FAM177A1 and ficolin-1 are generated from whole blood and function with the circulation of blood. Moreover, the result of CD40 (PP for H3 (PP3) =1) suggested that eQTLs and pQTLs have different signals in a single region. It is conceivable that CD40 could be generated from other tissues and leak into blood. Since the gene MANBA encoding beta-mannosidase cannot meet the single signal assumption (shown in the **Supplementary Figure**), the result of beta-mannosidase was excluded from our analyses. The detailed results are provided in ST12.

To eliminate potential LD, colocalization analysis between pQTLs and PBCs was conducted. As provided in **Table 2**, CD40 (PP4 = 0.977) and protein FAM177A1 (PP4 = 0.897) showed evidence for H4, indicating that they are likely to share a causal variant with PBC. As illustrated in **Figures 2C, D**, there is only one causal variant in a single region. This finding reinforced the protective effects identified in the MR analyses. It can be assumed that CD40 and protein FAM177A1 play an important role in the pathogenesis of PBC. They are potential drug targets for PBC. There is strong evidence (PP3 = 1.00) that beta-mannosidase and PBC have distinct causal variants in a gene region. This finding demonstrated that there could be a certain relationship between beta-mannosidase and PBC but not a causal relationship. The positive MR result of beta-mannosidase was affected by LD between the two variants. Therefore, beta-mannosidase was invalid and was excluded from our results. Since ficolin-1 did not meet the single causal variant assumption (shown in the **Supplementary Figure**), it was excluded from this study. Limited by the single signal assumption, we did not demonstrate shared variants between ficolin-1 and PBC. However, this did not indicate that the MR result of ficolin-1 is invalid, since it did not violate the three assumptions of MR.

**Table 2:** Colocalization result between significant proteins and PBC.

protein	beta-mannosidase	ficolin-1	CD40	protein FAM177A1
UniProtID	O00462	O00602	P25942	Q8N128
N	2436	1229	4199	3442
PP.H0	1.27×10^-120^	1.48×10^-97^	0	1.76×10^-134^
PP.H1	3.38×10^-17^	7.75×10^-01^	1.03×10^-02^	5.93×10^-03^
PP.H2	3.74×10^-104^	8.72×10^-99^	0	2.90×10^-133^
PP.H3	1	4.56×10^-02^	1.23×10^-02^	9.69×10^-02^
PP.H4	5.14×10^-10^	1.79×10^-01^	9.77×10^-01^	8.97×10^-01^

N is the number of SNPs in a single region. PP is the posterior probability for each hypothesis. H0: no causal variants for either trait. H1: a causal variant for trait 1. H2: a causal variant for trait 2. H3: two different causal variants for trait 1 and trait 2. H4: a shared causal variant between two traits. Beta-mannosidase showed a strong PP for H3, indicating that the positive result of beta-mannosidase was confounded by LD.

### Enrichment analysis

Based on the direction of the effect, causal proteins found in the discovery cohort were divided into risk and protective proteins. The enrichment analyses were conducted using two risk proteins and eight protective proteins respectively. Since FAM177A1 and FcR-like protein 3 were not found in the Reactome database, they were removed from the enrichment analysis. We found seven pathways enriched in risk proteins. Most of them were enriched in the two risk proteins simultaneously. Detailed results are provided in ST13. In the protective protein group, nine out of twenty-two pathways were overrepresented. They are closely related to complement activation, interleukin signaling and immunoregulatory interactions. It could be inferred that the disorders of these pathways could contribute to the incidence of PBC. Details of the significant pathways are provided in ST14.

## Discussion

In this study, to test the causal relationship between plasma proteins and PBC, we extracted 21,593 pQTLs for 2297 plasma proteins and conducted a large-scale proteome Mendelian randomization analysis. To avoid potential pleiotropy, we classified pQTLs into four different groups. The results of group A (sentinel cis-pQTLs only) were preferred. As mentioned previously, four circulating proteins (ficolin-1, beta-mannosidase, CD40, and protein FAM177A1) were identified as being associated with PBC in both the discovery cohort and replication cohort using univariable MR. To eliminate LD and examine the potential mechanisms, we conducted colocalization analyses on the proteins found in MR. The colocalization analysis showed that the result of beta-mannosidase was confounded by LD. Therefore, it was excluded from the results. Among the remaining three proteins, CD40 and protein FAM177A1 strongly colocalized with PBC. This further reinforced their causal effect on PBC. Furthermore, pQTLs of the proteins FAM177A1 and ficolin-1 colocalized with eQTLs of the genes FAM177A1 and FCN1 in whole blood, indicating that they were generated and functioned in whole blood.

Ficolin-1 is a kind of extracellular lectin that functions as a pattern-recognition receptor in innate immunity (32). It is secreted by monocytes/macrophages and granulocytes and exerts its function during inflammation. It has been demonstrated that ficolin-1 can directly facilitate the clearance of apoptosis (33). Furthermore, Ma et al. argued that ficolin-1 is one of the bridging molecules required for PTX3 to mediate the clearance of apoptotic cells (33). It is known that the ineffective clearance of apoptotic biliary epithelial cells (BECs), especially PDC-E2, is strongly correlated with the incidence of PBC (34). Consequently, it can be speculated that the lack of ficolin-1 could contribute to the development of PBC. A negative correlation between PBC and plasma ficolin-1 was also observed in this study. Consistent with our results, Hayashi et al. found that a low level of ficolin-1 dramatically increased the rate of developing liver cirrhosis in PBC patients independent of histological stage and ALP levels (35). However, an elevated level of circulating ficolin-1 in PBC patients was also observed by Hayashi (35). It could be assumed that the inflammatory condition facilitated the release of ficolin-1. Moreover, the low pH of infection conditions can cause a significant interaction between ficolin-1 and CRP (36). This interaction results in a decrease in the ficolin-1-GPCR43 interaction and downregulates IL-8 production (36). Therefore, the interaction could reduce inflammation by negative feedback regulation. This could explain why ficolin-1 is elevated in PBC patients. In addition, Brinkmann et al. found that ficolin-1 and ficolin-2 could mediate C3/C4 deposition onto mitochondria from serum, suggesting that ficolin may be involved in the homeostatic clearance of mitochondria released into the circulation (37). This evidence replicates the fact that the deposition of complement was found around the bile duct in PBC patients (38). In line with our enrichment results, five pathways related to complement activation were enriched for ficolin-1. Moreover, our colocalization results revealed strong evidence of colocalization between pQTLs and eQTLs of ficolin-1. Combined with the MR results, the colocalization corroborated that ficolin-1 is generated from blood and functions as a protective protein for PBC in the liver.

CD40 is a costimulatory member of the tumor necrosis factor receptor superfamily (39). There are two types of CD40: membrane CD40 (mCD40) and soluble CD40 (sCD40) (40). The interaction between mCD40 and CD40 ligand (CD40L) plays an important role in several autoimmune diseases, including rheumatoid arthritis, autoimmune nephritis, and PBC (39). Activation of CD40 was also found in PBC patients. Increased expression of CD40, Fas, and FasL in the bile ducts of PBC livers was observed by Afford et al. (41) They also found that the engagement of CD40 was a proapoptotic signal (41). CD40 on BECs activated by CD40L increased the transcriptional expression of FasL and induced apoptosis (41). In line with our enrichment analysis results, Afford et al. found that NF-kB was activated after CD40 ligation (41). The genes NFKB1 and TNFSF15 (which mediates the activation of NFKB and promotes apoptosis) were identified as risk loci in GWASs of PBC (42, 43). It is logical that the CD40/CD40L interaction is activated in PBC patients. However, a significant protective effect of circulating CD40 on PBC was found in our study. This could be attributed to the effect of sCD40, which is measured in plasma. sCD40 functions as a natural antagonist of CD40 by shedding the CD40/CD40L interaction (40). sCD40 is generated by alternative splicing (44). mCD40 cleaved by tumor necrosis factor-α-converting enzyme after its ligation with CD40L could also generate sCD40 (40). It could be concluded that both genetics and the increase in the CD40/CD40L interaction could elevate the level of sCD40, which could antagonize the CD40/CD40L interaction. Our colocalization result further revealed a strong probability of a shared causal variant between sCD40 and PBC. This finding can be explained by the excessive CD40/CD40L interaction and the simultaneous generation of sCD40. This result is consistent with the results of the MR analysis. Based on the results of the MR and colocalization analyses, it can be assumed that CD40 could be a drug target for PBC. Furthermore, CD40 was expressed in all antigen-presenting cells. As a result, this could be a reason why PBC might relapse after liver transplantation.

The protective effect of the protein FAM177A1 was found in our MR analysis. In line with previous colocalization analyses, it can colocalize with PBC and eQTLs in whole blood, suggesting that protein FAM177A1 was generated in whole blood and truly affected the pathogenesis of PBC (22). However, the biological function and characteristics of FAM177A1 remain unclear. The mechanisms underlying the relationship between FAM177A1 and PBC may be an interesting and challenging field for future analyses. Our study broadens the biomarkers for PBC, and future analyses should determine the underlying mechanism.

Although encouraging results were found, there are still drawbacks in our study. First, the pQTLs used in this analysis were obtained using two different platforms: Olink and Somascan. Although most proteins were obtained using Somascan, this could be a cause of heterogeneity. Second, although the sample size of the discovery cohort was sufficient to identify the potential causal genes, the number of cases in the FinnGen cohort was relatively small. Because only the participants of the FinnGen cohort did not overlap with the discovery cohort, the FinnGen cohort was the only choice for replication. If a significant protein discovered in the discovery cohort could be replicated in the FinnGen cohort, it suggests its robust causal effect on PBC. As a result, proteins identified in the discovery cohort and not replicated in the FinnGen cohort could also affect PBC. Third, in addition to the methods of colocalization adopted in this study, a more sophisticated method of colocalization based on the Sum of Single Effects regression (SuSiE) framework, which allows multiple signals in a single region, can be used. However, it is sensitive to the LD reference panel, and errors occurred when using the publicly available European LD reference panel. As a result, SuSiE was not adopted in this study. Moreover, the effect of four proteins was found in our analysis, yet the mechanisms of beta-mannosidase and protein FAM177A1 are unclear. Future work is needed. Finally, we discussed the relationships between plasma proteins and PBC. However, some proteins are expressed locally and are not secreted into the circulation. Therefore, future work should focus on proteins expressed in the liver that are related to PBC.

In general, we expand the current biomarkers for PBC and offer an understanding of the pathogenesis of PBC. In total, three (ficolin-1, CD40, and protein FAM177A1) plasma proteins were identified and replicated as being associated with PBC in MR analysis. All of them showed significant protective effects against PBC. CD40 and FAM177A1 share causal variants with PBC. These proteins can be potential biomarkers or drug targets for PBC.

## Data availability statement

The original contributions presented in the study are included in the article/**Supplementary Material**. Further inquiries can be directed to the corresponding author.

## Author contributions

HY and YL designed this research; HY did the data acquisition; HY and LC conducted the statistical analyses; HY wrote the first draft of the manuscript; LC and YL revised the manuscript. YL gave the final approval for the manuscript submission. All authors contributed to the article and approved the submitted version.
