# Supplementary material for: A large-scale plasma proteome Mendelian randomization study identifies novel causal plasma proteins related to primary biliary cholangitis
Source: Front Immunol. 2023 Feb 7;14:1052616. doi: 10.3389/fimmu.2023.1052616 (PMC9941641; doi:10.3389/fimmu.2023.1052616)
Supplement: Supplementary file 1 [file DataSheet_1.zip › supplementary figure.pdf]

Supplementary figures

SF1 Colocalization result between gene MANBA and beta-mannosidase
SF2 Colocalization between beta-mannosidase and PBC
SF3 Colocalization between ficolin-1 and PBC

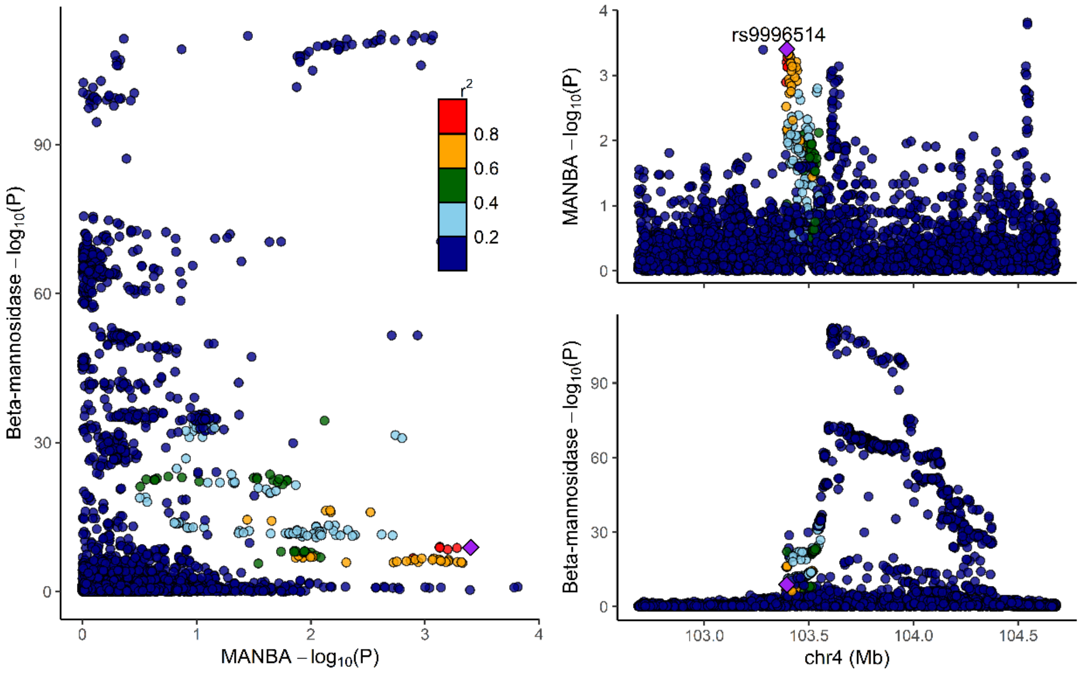

SF1 Colocalization result between gene MANBA and beta-mannosidase

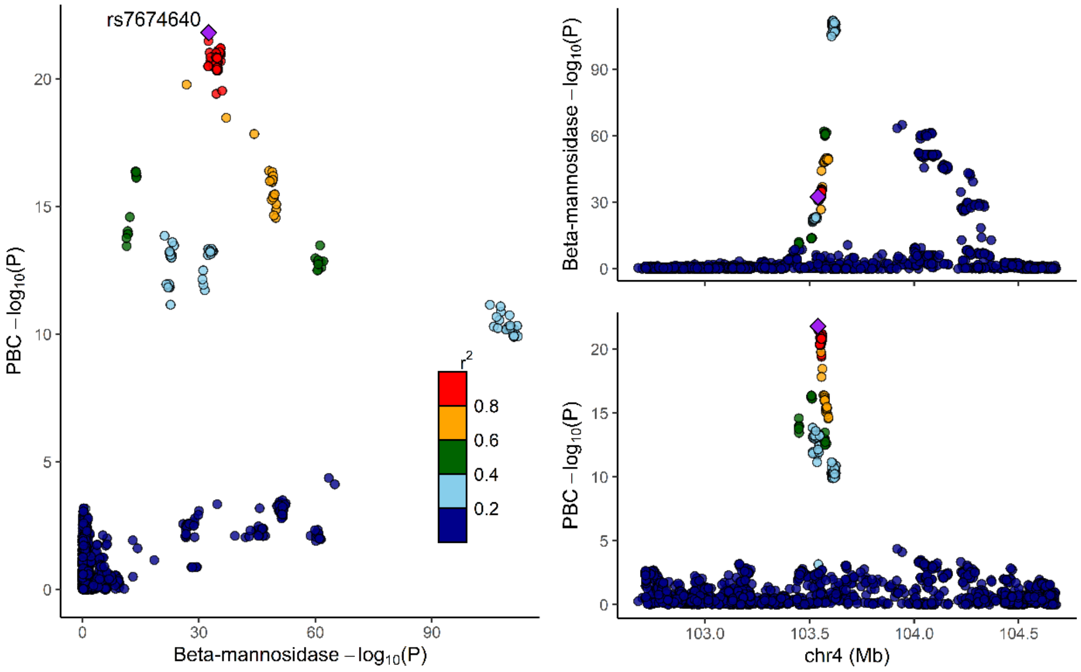

SF2 Colocalization between beta-mannosidase and PBC

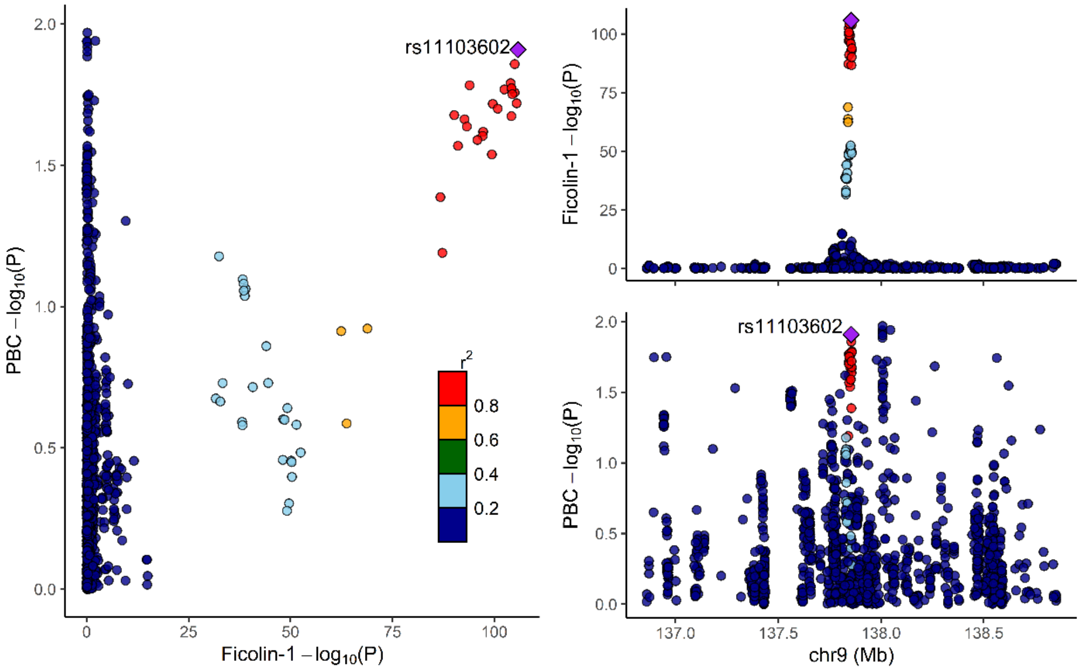

SF3 Colocalization between ficolin-1 and PBC
